# Dual task measures in older adults with and without cognitive impairment: response to simultaneous cognitive-exercise training and minimal clinically important difference estimates

**DOI:** 10.1186/s12877-023-04390-3

**Published:** 2023-10-16

**Authors:** I-Chen Chen, I-Ching Chuang, Ku-Chou Chang, Chih-Hung Chang, Ching-Yi Wu

**Affiliations:** 1 Department of Occupational Therapy, College of Nursing and Health Sciences, Da-Yeh University, Changhua, Taiwan; 2 Department of Occupational Therapy and Graduate Institute of Behavioral Sciences, College of Medicine, Chang Gung University, No.259, Wunhua 1st Rd., Gueishan Township, Taoyuan, 333 Taiwan; 3 Department of Neurology, Linkou Chang Gung Memorial Hospital, Taoyuan, Taiwan; 4 Department of Neurology, Kaohsiung Chang Gung Memorial Hospital, Kaohsiung, Taiwan; 5 Department of Medicine, College of Medicine, Chang Gung University, Taoyuan, Taiwan; 6 Program in Occupational Therapy, School of Medicine, Washington University , St. Louis, MO USA; 7 Department of Medicine, School of Medicine, Washington University , St. Louis, MO USA; 8 Department of Orthopedic Surgery, School of Medicine, Washington University , St. Louis, MO USA; 9 Healthy Aging Research Center, Chang Gung University, Taoyuan, Taiwan; 10 Department of Physical Medicine and Rehabilitation, Linkou Chang Gung Memorial Hospital, Taoyuan, Taiwan

**Keywords:** Dual task performance, Simultaneous cognitive-exercise training, Responsiveness, Minimal clinically important difference, Older adults, Cognitive impairment

## Abstract

**Background:**

Responsiveness and minimal clinically important difference (MCID) are critical indices to understand whether observed improvement represents a meaningful improvement after intervention. Although simultaneous cognitive-exercise training (SCET; e.g., performing memory tasks while cycling) has been suggested to enhance the cognitive function of older adults, responsiveness and MCID have not been established. Hence, we aimed to estimate responsiveness and MCIDs of two dual task performance involving cognition and hand function in older adults with and without cognitive impairment and to compare the differences in responsiveness and MCIDs of the two dual task performance between older adults with and without cognitive impairment.

**Methods:**

A total of 106 older adults completed the Montreal Cognitive Assessment and two dual tasks before and after SCET. One dual task was a combination of Serial Sevens Test and Box and Block Test (BBT), and the other included frequency discrimination and BBT. We used effect size and standardized response mean to indicate responsiveness and used anchor- and distribution-based approaches to estimating MCID ranges. When conducting data analysis, all participants were classified into two cognitive groups, cognitively healthy (Montreal Cognitive Assessment ≥ 26) and cognitively impaired (Montreal Cognitive Assessment < 26) groups, based on the scores of the Montreal Cognitive Assessment before SCET.

**Results:**

In the cognitively healthy group, Serial Seven Test performance when tasked with BBT and BBT performance when tasked with Serial Seven Test were responsive to SCET (effect size = 0.18–0.29; standardized response mean = 0.25–0.37). MCIDs of Serial Seven Test performance when tasked with BBT ranged 2.09–2.36, and MCIDs of BBT performance when tasked with Serial Seven Test ranged 3.77–5.85. In the cognitively impaired group, only frequency discrimination performance when tasked with BBT was responsive to SCET (effect size = 0.37; standardized response mean = 0.47). MCIDs of frequency discrimination performance when tasked with BBT ranged 1.47–2.18, and MCIDs of BBT performance when tasked with frequency discrimination ranged 1.13–7.62.

**Conclusions:**

Current findings suggest that a change in Serial Seven Test performance when tasked with BBT between 2.09 and 2.36 corrected number (correct responses – incorrect responses) should be considered a meaningful change for older adults who are cognitively healthy, and a change in frequency discrimination performance when tasked with BBT between 1.47 and 2.18 corrected number (correct responses – incorrect responses) should be considered a meaningful change for older adults who are cognitively impaired. Clinical practitioners may use these established MCIDs of dual tasks involving cognition and hand function to interpret changes following SCET for older adults with and without cognitive impairment.

**Trial registration:**

NCT04689776, 30/12/2020.

**Supplementary Information:**

The online version contains supplementary material available at 10.1186/s12877-023-04390-3.

## Introduction

It is estimated that 22% of the world’s population will be people aged 60 years and older by 2050 [1]. Age-related cognitive decline is frequently documented [2, 3], and may reduce the autonomy, functional independence, and quality of life of older adults and increase health care costs. A growing body of evidence suggests that simultaneous cognitive-exercise training (SCET; e.g., performing memory tasks while cycling) improves the cognitive function of older adults with [4–6] and without [6–11] cognitive impairment (CI). After an intervention, it is critical to quantify the amount of improvement that represents a meaningful change in comparison to a statistically significant change. Meaningful change allows clinical practitioners to assess and enhance cognitive abilities, as well as predict, prevent, and manage cognitive dysfunction in older adults. However, important psychometric indices have not been established for older adults following SCET.

Based on the guided plasticity facilitation framework, combined cognitive and physical training has been designed to enhance cognitive abilities [12]. The SCET program incorporates concurrent cognitive and physical components as demonstrated in studies, such as performing a maze task while cycling [8]. SCET has shown positive effects in preventing the progression from mild cognitive impairment to Alzheimer’s disease or other forms of severe cognitive impairment [4, 6, 13]. In cognitively healthy (CH) older adults, SCET has been implemented with various cognitive components paired with diverse physical components, resulting in improved cognitive abilities. For example, Theill et al. (2013) combined working memory and walking in the SCET intervention and observed enhanced executive functions in participants [9]. Wollesen et al. (2017) paired executive functions with balance training in SECT, leading to improvements in executive functions and balance performance [10].

Numerous studies on SCET have adopted multicomponent interventions, which have shown promising results in improving cognitive function in older adults with and without CI [4, 6, 13]. These multicomponent interventions typically involve various cognitive programs, encompassing attention, processing speed, memory, language, mental arithmetic, and executive functions. Additionally, they incorporate diverse physical programs, including aerobic, muscle-strengthening, balance exercises, and flexibility [7, 8, 14, 15]. The integration of these cognitive and physical components in SCET aims to improve different aspects of cognitive abilities in CH older adults. Studies utilizing such multicomponent approaches have demonstrated improvement in attention [7, 15], memory [7, 15, 16], language [15], executive functions [5, 8, 16, 17], as well as enhanced physical function [5, 7, 14, 18]. Furthermore, several studies have demonstrated the positive effects of SCET on dual task performance in CH older adults [7–10, 14, 18].

Dual task paradigm is a procedure in which an individual is required to simultaneously perform two tasks, and each task involves a distinct goal [19, 20]. Previous studies have primarily focus on dual task walking performance in older adults following SCET [7–11, 14, 18, 21, 22]. However, it is important to note that dual task involving both cognition and hand function are pervasive in various real-world activities. For instance, counting (backward) while cutting food or listening to the news while brushing their teeth. Previous observational studies examining dual tasks involving cognition and finger-tapping tasks in older adults have primarily focused on investigating the dual task costs [23–25]. Acaröz Candan and Özcan (2019) suggest that dual task performance involving cognition and hand function could serve as a predictor of activities of daily living performance [26]. However, there is a noticeable gap in research concerning the training effect of SCET, specifically targeting dual task performance involving cognition and hand function in older adults.

Cognition in dual task performance involves executive functions (EFs), a collection of top-down cognitive control processes [27]. EFs allocate and schedule the limited cognitive resources [28–31], which enables people to perform two or multiple tasks simultaneously [27]. Previous intervention studies assessed EFs of dual task performance using paper-pencil tests such as the Trail Making Test [15, 32], neuropsychological tests such as the Stroop Color-Word Test [10, 17, 33], or laboratory-based assessments such as Go/No-Go Test [32, 34]. These measures are less ecologically valid compared to having individuals perform a dual task that closely resembles an everyday task they would typically encounter. While dual tasks involving walking are commonly used, they carry a risk of falling and may be limited by space constraints. On the other hand, dual tasks involving hand function pose no risk of falling and are less space restriction. Furthermore, we intended to establish a connection between the dual task measure and daily dual task performance. By incorporating tasks such as grasping/moving, mental calculation, and hearing discrimination commonly encountered in daily life, researchers can gain insights into the cognitive-motor integration required for everyday functioning. Grasping/moving plays a crucial role in our everyday activities and is essential for performing various tasks. For instance, grasping a cup of tea, using a knife while preparing meals, and grasping a doorknob to open a door. Mental calculation skills are particularly valuable when we go shopping. Mental calculations enable us to manage our finances and make informed purchasing choices. Hearing discrimination allows us to distinguish between informative and non-informative sounds and to focus on relevant information. This skill is essential for responding appropriately to alarms, notifications, or important auditory cues in our surroundings. Understanding how individuals perform cognitive and hand function tasks simultaneously provides valuable information about functional capacities and independence in older adults.

To assess meaningful and observed changes following an intervention, the degree of beneficial effect must be determined and is usually indicated by responsiveness and minimal clinically important difference (MCID). Responsiveness is defined as the ability of a measure to detect a change in a construct that is being assessed over a specific period [35, 36]. MCID is defined as “the smallest difference in score in the domain of interest which patients perceive as beneficial and which would mandate, in the absence of troublesome side effects and excessive cost, a change in the patient’s management [29, p.408].” MCID determination can be broadly categorized into two methods: distribution-based and anchor-based. The distribution-based method is based on the statistical properties of an observed sample to reflect changes following interventions. Anchor-based method uses an external criterion that is moderately correlated with the estimated assessment [37–40]. Although the distribution-based approach can identify changes that are not likely to be attributed to random measurement variation, it may lack direct clinical relevance. Conversely, the anchor-based approach can establish changes that are clinically relevant, but it depends on an external anchor [40, 41]. Relying solely on one approach for MCID estimation could be limiting, as each approach has its inherent weaknesses in the methodology. Therefore, prior research suggests that utilization of multiple approaches and the reporting of a narrow range of MCID estimates [39, 40].

While there have been studies that examine dual task walking performance after training, no studies have estimated the responsiveness and MCID of dual tasks involving hand function or walking post-intervention in any population. On the other hand, MCID estimates of a measure may vary across populations with differing levels of cognitive function [42]. To account for these distinctions, distinct MCID estimates are necessary for individuals who are CH and those who have CI. This study identified CH and CI populations based on participants’ performance on Montreal Cognitive Assessment (MoCA).

The aims of this study were to (1) establish the responsiveness and MCID of dual task performance involving cognition and hand function for older adults with and without CI following SCET, and (2) compare the differences in responsiveness and MCID of dual task performance involving cognition and hand function between older adults with and without CI following SCET.

## Methods

### Participants

This study is an ongoing clinical trial study, and the study protocol for this intervention has been approved by the local institutional review board (#201912EM016). Participants’ inclusion criteria were (1) age of 60 years or over, (2) able to follow instructions (Mini-Mental State Evaluation ≥ 20), (3) without difficulties in basic activities of daily living, (4) self- or informant-report cognitive complaints, and (5) without a diagnosis of dementia by physicians. Participants who were diagnosed with neurological disorders or having an unstable medical condition (e.g., recent myocardial infarction, heart failure, recent heart surgery, or severe asthma) were excluded from this study.

### Procedure

All participants were invited from adult day care centers or community facilities and were screened to ensure their eligibility for this study. After participants provided written informed consent, they participated in SCET and completed measures before and after SCET. Research assistants were responsible for implementing all measures in this study. To minimize potential confounding effects, the measures conducted before and after SCET were scheduled on different days and did not occur on the same day as the SCET. This approach ensured that the assessments were conducted independently and allowed for a precise evaluation of the effects of the SCET intervention.

Instructors who conducted the intervention were qualified occupational therapists. To minimize the staff training weakness because of multi-site sampling, all instructors received standardized training on the concepts of SCET. Instructors were not involved in the assessments and were blind to the cognitive status of all participants. Their lack of involvement in the assessments ensured objectivity and minimized potential biases that could arise from their knowledge of the participants’ cognitive level.

**Fig. 1:**
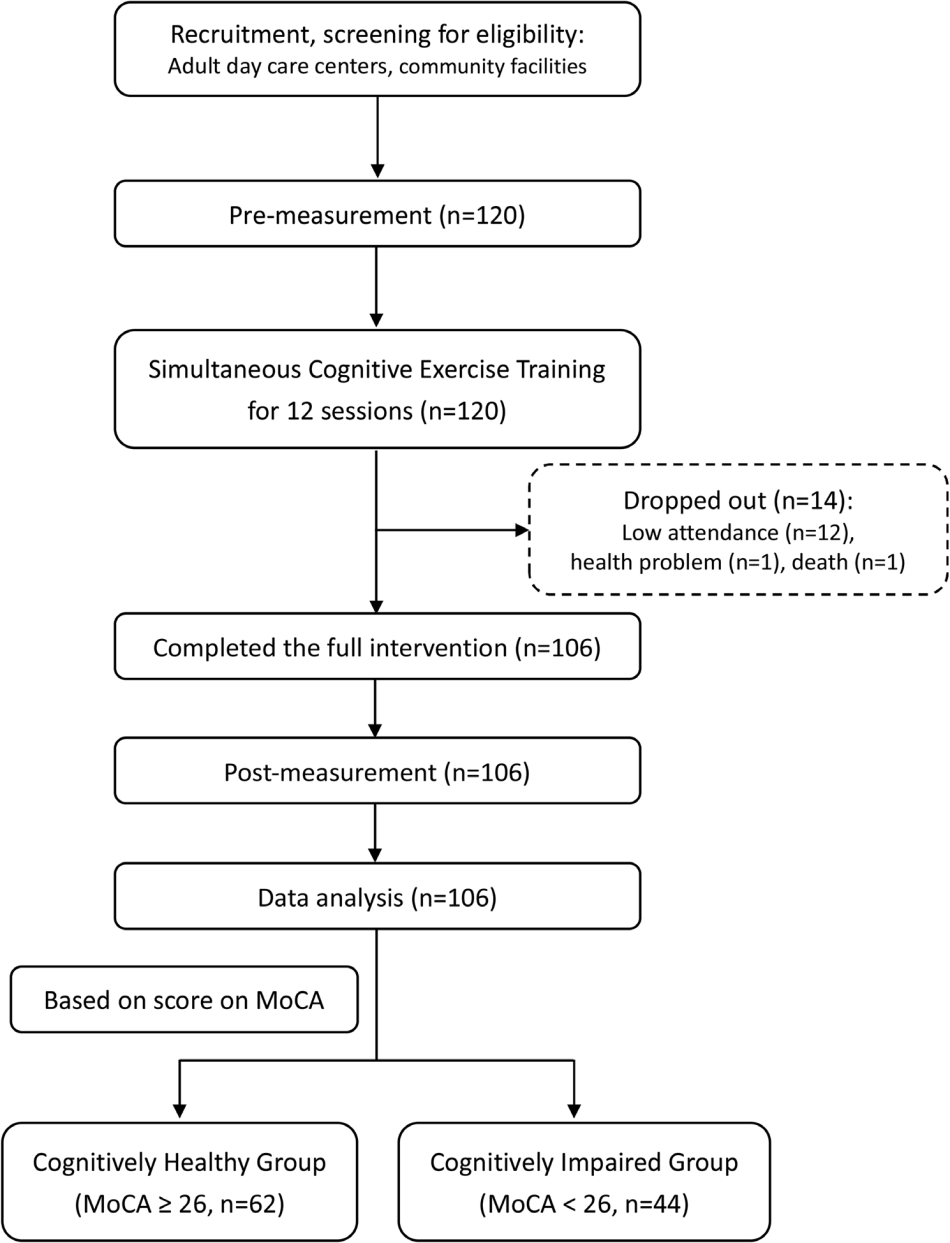
Flow diagram of screening, intervention, measurement, and final sample included in data analysis

At the data analysis stage, all participants were divided into two cognitive groups based on their performance on MoCA before SCET, CH (MoCA ≥ 26), and CI (MoCA < 26) [43] for additional analysis. Participants who missed more than 30% of sessions were excluded from the data analysis (Fig. 1).

### Intervention

The SCET was developed as an arm of a larger project focused on combined training for older adults. The overarching goal of SCET was to improve cognitive function. Participants were instructed to concurrently perform cognitive and exercise tasks, with greater emphasis on cognitive components. It was led by qualified occupational therapists who served as in-person instructors. Throughout the SCET intervention, participants had the opportunity to receive feedback from the in-person instructor. The program consisted of 90–120 min, once a week, for 12 weeks in a group-based setting.

**Fig. 2:**
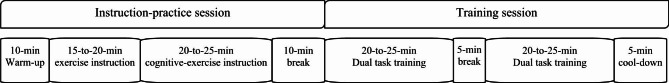
Time structure of the SCET intervention

Each session was divided into two parts: instruction-practice and training sessions. The instruction-practice session began with a warm-up (10 min), followed by exercise activities (15–20 min). Subsequently, cognitive components were introduced and integrated (20–25 min). The primary aim of this session was to familiarize participants with the simultaneous performance of cognitive and exercise tasks. Following a 10-minute break, the training session commenced. Initially, dual task training was performed for 20–25 min, followed by a short 5-min break, and then another 20–25 min of dual task training. The session concluded with a 5-minute cool-down stretching routine. The primary aim of this session was to make participants implement dual tasks they had learned during the instruction-practice session and actively engaged in dual task training. The time structure of the SCET intervention is illustrated in Fig. 2.

Each SCET session encompassed cognitive and physical components.

The cognitive components consisted of attention, processing speed, memory, calculation, and language. Each session incorporated one or more of these cognitive components. Attention activities involved tasks such as responding to visual or auditory cues/instructions as well as identifying or differentiating colors, shapes, amounts, or sizes of pictures. Processing speed activities required participants to answer questions or follow visual or verbal instructions within a limited time period. Memory activities involved repeating a series of digits or words after seeing or hearing them, performing a series of visual or verbal instructions after seeing or hearing them, and remembering visual images, shapes, or colors. Calculation tasks included answering arithmetic questions presented on the screen or determining the total amount of items taken from the simulated shop or a shopping list. Language tasks encompassed naming fruits, animals, places, or objects in specific environments, as well as constructing logical stories based on a series of given pictures.

The physical components of the program consisted of stretching, aerobic exercise, muscle-strengthening exercise, and balance exercise. Participants were encouraged to exercise at moderate intensity, targeting 50–70% of their maximum heart rate. The warm-up and cool-down included upper and lower limb stretching. Aerobic activities involved walking, marching on the spot, and tapping the floor with toes or heels. Balance activities included standing with a narrow base, cross-stepping, and kicking a ball. Muscle-strengthening exercises involved carrying objects and performing upper limb workouts with weighted bottles (filled with water) (e.g., bicep curls, overhead triceps press), as well as lower limb workouts with or without elastic bands (e.g., squats, lunges).

During the SCET, attention, processing speed, memory, calculation, and language tasks were performed simultaneously with aerobic, muscle-strengthening, and balance exercises. Stretching exercises were integrated into the warm-up and cool-down portions of the session and were not paired with specific cognitive components. The pairings of cognitive and exercise tasks varied throughout the intervention, with cognitive and physical demands progressively increasing over trials. For instance, the number of words to be recalled or the resistance of the elastic bands used in exercises increased throughout the program. The adaptation of cognitive task difficulty was based on participants’ performance in the previous session. In cases where the cognitive component’s difficulty level was augmented, the difficulty level of the corresponding physical component remained constant. This approach was adopted to prevent potential adverse effects on participants’ performance, ensuring they were not overwhelmed by the task requirements. However, given the group-based training, the adjusted difficulty level of cognitive tasks may not have been perfectly tailored to each participant’s individual capabilities.

### Measures

The MoCA was used to assess global cognition and was chosen as the external criterion for anchor-based MCID estimates. Two dual tasks were used to assess dual task performance.

#### MoCA

The MoCA is a sensitive and validated tool [44, 45] to assess an individual’s global cognition. The MoCA consists of 12 items to evaluate orientation to time and place, attention, concentration, short-term memory, working memory, visuospatial abilities, language, and EFs. An individual’s level of education is taken into consideration. If an individual has 12 years or less of formal education, 1 point is added to the total score. The total score ranges from 0 to 30, and a higher score indicates better global cognition. A total score of 26 or above is regarded as normal cognitive function and a total score of 19 to 25 indicates mild cognitive impairment [43]. In this study, we used the Taiwanese Version of the MoCA [46].

#### Dual tasks

The two dual tasks that we designed in this study were: (1) Serial Seven Test (SST) and Box and Block Test (BBT; SST and BBT performed concurrently for 60 s) and (2) frequency discrimination (FD) and BBT (FD and BBT performed currently for 60 s).

The SST is intended to evaluate working memory [47]. The BBT is designed to evaluate an individual’s gross manual dexterity [48]. The FD is designed to evaluate sustained attention [49, 50].

SST: a participant is asked to repeatedly count backward by seven which begins with 300 (i.e., subtract 7 from 300), and to report their answer to each subtraction. Correct and incorrect responses to SST are recorded, and the corrected number which is the difference between correct and incorrect responses is calculated.

BBT: a participant is seated at a table and presented with a rectangular box divided into two square compartments and there are 150 colored wooden blocks in one compartment. A participant is instructed to move a block, one at a time, from one compartment to the other as many as they can in 60 s [48]. The number of blocks that are moved from one compartment to another compartment is recorded.

FD: a participant is required to discriminate between either high (1000 Hz) or low (500 Hz) pitch presented by speakers and to report their answer. There was a total of 18 trials conducted in 60 s. Specifically, there were nine trials involving high-pitch sounds and nine trials involving low-pitch sounds. The presentation of both high- and low-pitch sounds was randomized, and the intervals between the sounds ranged randomly from 1 to 4 s. Correct and incorrect responses to FD are recorded, and the corrected number which is the difference between correct and incorrect responses is calculated.

We focused on dual task performance and each dual task included cognitive and physical performance. We reported both cognitive and physical performance for each dual task. SST performance when tasked with BBT indicated the cognitive performance, the corrected number of SST, in the dual task of SST and BBT. BBT performance when tasked with SST indicated the physical performance, the number of blocks, in the dual task of SST and BBT. FD performance when tasked with BBT indicated the cognitive performance, the corrected number of FD, in the dual task of FD and BBT. BBT performance when tasked with FD indicated the physical performance, the number of blocks, in the dual task of FD and BBT.

Before engaging in dual tasks, participants had the opportunity to practice SST, FD, and BBT under single task conditions. Research assistants provided instructions on how to perform the dual tasks, and participants were given two practice sessions to ensure a comprehensive understanding of how to successfully perform the dual tasks before undergoing evaluation.

Near and far transfer refers to the extent to which the benefits of training on a specific task can be applied to other tasks. Near transfer occurs when skills gained from a training task can be readily applied to tasks with similar processes or demands. It typically occurs when the training and the transfer tasks are closely related. On the other hand, far transfer involves the generalization of learning to tasks with dissimilar processes and demands. In this case, the acquired skills are applied to domains that may not share obvious similarities with the trained task [51, 52].

The cognitive components of SCET included attention and calculation tasks. The attention activities involved various tasks, such as responding to visual or auditory cues and instructions. FD required participants to determine high- or low-pitch sounds, which showed some similarity to responding to auditory cues, making it a relatively near transfer task. Similarly, the calculation activities involved answering arithmetic questions and determining the total amounts. SST required participants to perform serial subtraction by 7, mental arithmetic, making it another relatively near transfer task. Regarding the physical components, participants engaged in muscle-strengthening exercises by grasping bottles filled with water as weights or using elastic bands. While grasping was incorporated into both BBT and SCET, the patterns of grasping blocks and bottles/elastic bands were not quite similar. As a result, BBT was considered a relatively far transfer task.

### Data analysis

Descriptive statistics were used to summarize the demographic characteristics of all participants. We used the standardized response mean (SRM) and effect size (ES) to evaluate the responsiveness of the two groups on two dual task performance. The formula for these statistics are as follows, where D = raw score change on measure between pre- and post-intervention; SD = standard deviation at pre-intervention; SD_c_ = standard deviation of D; ES = D/SD; SRM = D/SD_c_ [36, 53]. According to Cohen’s guideline [54] for interpreting the magnitude of responsiveness, where ES/SRM = 0.2 is small, ES/SRM = 0.5 is medium, and ES/SRM = 0.8 is large.

We used distribution-based and anchor-based approaches to estimate the MCID of dual task measures. Distribution-based MCID is calculated as one-half of the SD of all participants at pre-intervention [55]. For the anchor-based approach, we selected MoCA as the external criterion. As the MoCA showed medium-to-large correlations with two dual task measures (ρs > 0.26, *p*s < 0.001), the MoCA is an appropriate criterion measure in this study. The anchor-based MCID is calculated as the average score change of participants whose score change is equal to or greater than one-half of the SD of MoCA at pre-intervention [56].

## Results

### Participants

Initially, 120 eligible participants were recruited in this study and participated in SCET. Of these, 14 were excluded due to poor attendance < 70%, and 106 participants completed pre- and post-intervention measures and were included in data analysis. Four of the 106 participants were diagnosed with arthritis, and 3 of the 4 were taking medication for arthritis. SST performance when tasked with BBT and FD performance when tasked with BBT were significantly improved in all participants who participated in SCET (SST: *t* = 2.43, *p* = 0.017; FD: *t* = 3.21, *p* = 0.002); however, all participants had similar BBT performance when tasked with SST and with FD at pre- and post-intervention (*t*s < 1.55, *p*s > 0.12). According to the scores of MoCA at pre-intervention, all participants were grouped into CH (MoCA ≥ 26, n = 62) and CI (MoCA < 26, n = 44) groups. Participant characteristics are reported in Table 1.

**Table 1:** Participant characteristics

	All participants (n = 106)		Cognitive Health (n = 62)		Cognitive Impairment (n = 44)	
	*M*	(*SD*)	*M*	(*SD*)	*M*	(*SD*)
Age (years)	71.56	(6.65)	69.71	(6.24)	74.17	(6.38)
Sex (female; n[%])	90	(84.91)	55	(87.30)	35	(79.55)
Education (years)	9.46	(3.92)	10.96	(3.03)	7.34	(4.08)
MMSE (points)	27.43	(2.55)	28.65	(1.46)	25.73	(2.78)
MoCA (points)	25.38	(4.34)	28.15	(1.38)	21.48	(4.09)

Note. Education, years of formal education. MMSE, Mini-Mental Status Examination; MoCA, Montreal Cognitive Assessment. Based on the result of independent *t*-test, there was a significant difference in years of formal education between two groups (*t* = 5.243, *p* < 0.001)

### Responsiveness

Table 2 shows the responsiveness of the two dual task measures to SCET. In the CH group, two dual task measures were responsive to SCET, with small ES and SRM values (ESs = 0.18–0.29; SRMs = 0.20–0.37), except for BBT performance when tasked with FD (ES = 0.08; SRM = 0.10). In the CI group, FD performance when tasked with BBT was responsive to SCET, with small-to-medium values of ES and SRM (ES = 0.37; SRM = 0.47).

**Table 2:** Responsiveness to simultaneous cognitive-exercise training across two groups

	Cognitive Health (n = 62)		Cognitive Impairment (n = 44)	
Measures	ES	SRM	ES	SRM
SST performance when tasked with BBT (Crt No)	0.29	0.37	0.04	0.03
BBT performance when tasked with SST (No)	0.18	0.25	0.02	0.03
FD performance when tasked with BBT (Crt No)	0.22	0.20	0.37	0.47
BBT performance when tasked with FD (No)	0.08	0.10	-0.01	-0.02

Note. ES, effect size; SRM, standardized response mean; Crt No, corrected number = correct responses -incorrect responses; No, number of blocks

### MCID

The anchor-based MCID was calculated as the average score change of participants whose improved score was equal to or greater than one-half of the SD of MoCA at pre-intervention. One-half of the SDs of MoCA were 0.69 and 2.05 for the CH and the CI groups, respectively (Table 3). Since partial points on the MoCA are not possible, we rounded up the values of the criterion to 1 and 2 points, respectively. For the CH group, the anchor-based MCID was calculated as the average change scores of 23 participants with a score change of 1 or more. For the CI group, it was calculated as the average change scores of 16 participants with a score change of 2 or more. Among participants in the CH group, 37% (n = 23) demonstrated a meaningful improvement in MoCA scores from baseline. Of the participants in the CI group, 36% (n = 16) exhibited a meaningful increase in MoCA scores from baseline. Table 4 displays the distribution- and anchor-based and MCID estimates for the two groups.

**Table 3:** Dual-task measures across two groups

	Cognitive Health (n = 62)								Cognitive Impairment (n = 44)							
	Distribution-based approach (n = 62)				Anchor-based approach (n = 23)				Distribution-based approach (n = 44)				Anchor-based approach (n = 16)			
	Pre-intervention		Post-intervention		Pre-intervention		Post-intervention		Pre-intervention		Post-intervention		Pre-intervention		Post-intervention	
Measures	*M*	(*SD*)	*M*	(*SD*)	*M*	(*SD*)	*M*	(*SD*)	*M*	(*SD*)	*M*	(*SD*)	*M*	(*SD*)	*M*	(*SD*)
SST performance when tasked with BBT (Crt No)	7.25	(4.73)	8.37	(5.25)	7.45	(5.52)	9.39	(6.11)	1.59	(2.49)	1.63	(3.20)	2.75	(2.65)	3.33	(3.85)
BBT performance when tasked with SST (No)	55.51	(11.71)	57.53	(11.49)	52.59	(11.30)	56.74	(12.16)	45.14	(15.38)	46.50	(14.32)	52.56	(12.61)	52.47	(14.99)
FD performance when tasked with BBT (Crt No)	15.79	(2.41)	16.29	(2.55)	16.00	(1.88)	17.04	(1.46)	13.60	(4.36)	15.46	(2.60)	14.00	(2.58)	15.60	(2.13)
BBT performance when tasked with FD (No)	68.34	(11.97)	69.10	(10.88)	68.50	(11.77)	69.04	(11.51)	58.81	(15.23)	59.17	(13.15)	62.25	(15.65)	64.33	(11.91)
MoCA	28.15	(1.38)	28.27	(1.42)	27.35	(1.07)	29.00	(1.04)	21.48	(4.09)	21.59	(4.66)	22.31	(3.75)	25.13	(3.50)

Note. M, mean; SD, standard deviation; MoCA, Montreal Cognitive Assessment; Crt No, corrected number = correct responses - incorrect responses; No, number of blocks

**Table 4:** Distribution- and anchor-based MCID of the two dual-task measures across two groups

	Cognitive Health (n = 62)		Cognitive Impairment (n = 44)	
Measures	Distribution-based MCID (n = 62)	Anchor-based MCID (n = 23)	Distribution-based MCID (n = 44)	Anchor-based MCID (n = 16)
SST performance when tasked with BBT (Crt No)	2.36	2.09	1.24	0.67
BBT performance when tasked with SST (No)	5.85	3.77	7.69	1.07
FD performance when tasked with BBT (Crt No)	1.20	1.00	2.18	1.47
BBT performance when tasked with FD (No)	5.99	0.32	7.62	1.13

Note. Crt No, corrected number = correct responses - incorrect responses; No, number of blocks

## Discussion

To the best of our knowledge, this study is the first to estimate the responsiveness and MCID of dual task performance involving cognition and hand function for older adults with and without CI after SCET. Among the sample of CH adults, there were small yet consistent intervention effects to the cognitive components of different dual task paradigms; the effect on the exercise task was stronger when paired with the cognitive mental arithmetic task than when paired with cognitive auditory discrimination task.

This study suggests that the dual tasks combining cognition and hand function in the CH group show fair responsiveness to the SCET intervention. In the CH group, the dual task measures showed small responsiveness, except BBT performance when tasked with FD; in the CI group, only FD performance when tasked with BBT showed small-to-medium responsiveness. Both dual tasks may represent different levels of difficulty in successful execution. The dual task of SST and BBT required higher cognitive demand that SST involves working memory [47], a higher-order cognitive ability, and was more challenging. Regarding the other dual task of FD and BBT, FD involves sustained attention [49, 50], which is a fundamental cognitive ability [57, 58].

For the CH group, there may be a ceiling effect [59, 60] on BBT performance when tasked with FD. The average performances of the right hand of females and males aged 70–74 years on the BBT in 60 s under single task conditions were 68.6 and 66.3, respectively [48]. Participants in the CH group who had a high baseline BBT performance when tasked with FD showed limited room for improvement (mean of age = 69.71; means of BBT performance when tasked with FD at pre- and post-intervention = 68.34 and 68.50, respectively). The limited improvement may result in changes that are not easily noticeable. As a result, future studies are encouraged to explore dual tasks with more challenging physical tasks for participants in the CH group.

For participants in the CI group, the dual task of SST and BBT may be too challenging to be responsive to change and a floor effect [61] in the CI group might be found. The average SST performance when tasked with BBT at pre- and post-intervention were 1.59 and 1.63, respectively. In contrast, in the CH group, the average SST performance when tasked with BBT at pre- and post-intervention were 7.25 and 8.37, respectively. FD performance when tasked with BBT attained small-to-moderate responsiveness, which may be due to the improved performance and the decreased variability after intervention (pre-intervention: mean = 13.60, SD = 4.36; post-intervention: mean = 15.46, SD = 2.60). On the other hand, the great heterogeneity among individuals due to CI (i.e., great SD and SD_c_) may have weakened the responsiveness (ES = D/SD; SRM = D/SD_c_) in the CI group.

The estimates of distribution-based MCID are greater than those of anchor-based MCID of all dual task measures in both groups (distribution-based MCIDs: CH group: 1.20–5.99; CI group: 1.24–7.69; anchor-based MCIDs: CH group: 0.32–3.77; CI group: 0.67–1.47). The observed discrepancy between the two methods may be due to the different ways in which changes are detected. The distribution-based approach measures changes by calculating the statistical properties of the observed sample, including participants who improved or declined after the intervention. The anchor-based approach uses an external criterion is used as a reference for measuring changes [37–40] based on the performance of participants with improved global cognition in this study. According to MoCA scores, 37% of participants in the CH group and 36% in the CI group exhibited meaningful improvements, meeting the criteria for inclusion in the anchor-based approach. Despite variations in the criteria for meaningful change between the two groups, the percentages of participants in both groups who benefited from SCET and demonstrated substantial improvement were comparable. As recommended by previous studies [39, 40], we reported arrow ranges of MCID estimates derived from a combination of distribution-based and anchor-based approaches as our MCID estimates for dual task measures in older adults with CH and CI.

Our findings indicate that SST performance when tasked with BBT may serve as a MCID index for the CH group following SCET, while FD performance when tasked with BBT may serve as a MCID index for the CI group following SCET. Participants in the CH group achieved meaningful change along with a greater MCID estimate of SST performance when tasked with BBT (MCIDs = 2.09–2.36) than those in the CI group (MCIDs = 0.67–1.24). As noted earlier, the dual task of SST and BBT requires higher cognitive demand and is more complex than the dual task of FD and BBT. Accordingly, the findings suggest that participants in the CH group had better cognitive abilities and potential for improvement in the progress of cognitive performance and may have greater potential improvement from the SCET compared to those in the CI group. In terms of FD performance when tasked with BBT, participants in the CI group achieved meaningful change along with a greater MCID estimate (MCIDs = 1.47–2.18) than those in the CH group (MCIDs = 1.00-1.20). It is possible that the CI group had worse baseline FD performance when tasked with BBT (mean = 13.60) and more room to be improved than those in the CH group (mean = 15.79) when performing the easier dual task requiring the fundamental cognitive ability of sustained attention.

Given the small responsiveness of BBT performance when tasked with SST and with FD and the huge ranges between distribution- and anchor-based MCID estimates for both groups, BBT performance when tasked with SST and with FD were not suggested to be used as a MCID index after SCET for either group. Also, considering that the SCET intervention did not demonstrate significant improvements in BBT performance among older adults with CI, it may be worthwhile to explore alternative physical tasks such as marching on the spot or performing upper/lower limb workouts with resistance in future dual task paradigms for this specific population.

The results of the 12-week SCET intervention in a group-based setting, combining various cognitive tasks with multiple exercises for a total dose of 1080–1440 min, indicate improvements in attention (FD) and working memory (SST) performance during dual task conditions, as well as EFs (dual task performance) in CH older adults. These findings are aligned with previous SCET studies that employed multicomponent cognitive training (involving attention, memory, arithmetic, and language) combined with physical exercises (aerobic and resistance exercises) and reported improved attention and working memory performance in CH older adults [5, 7, 8, 15]. Regarding dual task targeting EFs, prior studies have also suggested SCET with single (memory and cycling) or multiple (attention, language, and mental calculation paired with aerobic and muscle-strengthening exercises) components can have positive effects on EFs in CH older adults [9, 17, 62].

In the CI group, attention showed improvement, which is consistent with previous research using multicomponent SCET [63, 64]. However, SCET intervention had no effect on working memory and EFs. Prior studies have indicated that multicomponent SCET with a longer duration of 24 weeks enhance working memory [64, 65] and EFs [65] in older adults with CI. This suggests that older adults with CI may require a higher intervention dosage with a longer duration to achieve improvements.

### Study limitations

This study has some limitations that should be noted. Firstly, the sample size was small, and there was an uneven distribution of sexes, which may affect the generalizability of our results to other populations. The psychometric properties vary, which depends on participant characteristics. Therefore, our findings are more properly generalizable to community-dwelling female older adults with or without CI than other populations. Secondly, only participants who attended more than 70% of the sessions were included, limiting the generalizability to those with high motivation for participation. Thirdly, the participants’ medical conditions were collected through self- or informant-report. Based on this information, participants with a diagnosis of hearing loss or hard of hearing were excluded from the study. However, no formal assessment was conducted to determine the hearing status of the participants. Fourthly, we did not consider the significant difference in years of formal education between the two groups when analyzing dual task performance, as we did with the MoCA score. Education level can influence cognitive abilities and task performance, and it should have been considered to control for potential confounding effects. Lastly, participants were grouped into two categories based on their pre-intervention MoCA score, but CI is diverse and can have varying levels. Future research with a larger sample size should explore psychometric properties for individuals with different levels of CI.

## Conclusions

This study suggests that when evaluating the benefits of SCET for CH older adults and those with CI using responsiveness and MCID of dual tasks involving cognition and hand function, SST performance when tasked with BBT and FD performance when tasked with BBT should be used as indices, respectively. A change in SST performance when tasked with BBT between 2.09 and 2.36 corrected numbers is considered meaningful for CH older adults. In comparison, a change in FD performance when tasked with BBT between 1.47 and 2.18 corrected numbers is considered a meaningful change for CI older adults.

### Electronic supplementary material

Below is the link to the electronic supplementary material.

Supplementary Material 1

## Data Availability

The datasets used and/or analyzed during the current study are available from the corresponding author on reasonable request.
