# Supplementary material for: Dual task measures in older adults with and without cognitive impairment: response to simultaneous cognitive-exercise training and minimal clinically important difference estimates
Source: BMC Geriatr. 2023 Oct 16;23:663. doi: 10.1186/s12877-023-04390-3 (PMC10580601; doi:10.1186/s12877-023-04390-3)
Supplement: Supplementary file 1 — Supplementary Material 1 [file 12877_2023_4390_MOESM1_ESM.docx]

Supplementary

**Table S1.** Participants characteristics of participants who dropped out

|  | All participants (n=14) | | Cognitive Health (n=8) | | Cognitive Decline (n=6) | |
| --- | --- | --- | --- | --- | --- | --- |
|  | *M* | (*SD*) | *M* | (*SD*) | *M* | (*SD*) |
| Age (years) | 70.06 | (6.16) | 68.42 | (6.84) | 72.24 | (4.79) |
| Sex (female; n[%]) | 14 | (100) | 8 | (100) | 6 | (100) |
| Education (years) | 8.93 | (4.68) | 10.88 | (4.82) | 6.33 | (3.20) |
| MMSE (points) | 27.14 | (2.63) | 28.63 | (1.41) | 25.17 | (2.64) |
| MoCA (points) | 24.71 | (3.27) | 27.13 | (0.83) | 21.5 | (2.26) |

*Note*. Education, years of formal education. MMSE, Mini-Mental Status Examination; MoCA, Montreal Cognitive Assessment.

**Table S2**. Comparisons between CH and CD groups at pre-intervention

|  | Cognitively Healthy (n=62) | Cognitive Decline (n=44) |  |  |
| --- | --- | --- | --- | --- |
| Measures | *M* (*SD*) | *M* (*SD*) | *t* | *p* |
| SST when tasked with BBT | 7.25 (4.73) | 1.59 (2.49) | 7.04 | < 0.001 |
| BBT when tasked with SST | 55.51 (11.71) | 45.14 (15.38) | 3.88 | < 0.001 |
| FD when tasked with BBT | 15.79 (2.41) | 13.60 (4.36) | 3.27 | 0.001 |
| BBT when tasked with FD | 68.34 (11.97) | 58.81 (15.23) | 3.55 | 0.001 |
| MMSE | 28.65 (1.46) | 25.73 (2.78) | 7.02 | < 0.001 |
| MoCA | 28.15 (1.38) | 21.48 (4.10) | 11.93 | < 0.001 |
| Digit-Symbol Coding | 12.98 (2.55) | 10.37 (2.48) | 5.58 | < 0.001 |
| Word List |  |  |  |  |
| Immediate recall | 12.94 (2.64) | 9.16 (2.48) | 7.44 | < 0.001 |
| Delayed recall | 12.53 (3.00) | 9.48 (2.23) | 5.72 | < 0.001 |
| Stroop Color and Word Test |  |  |  |  |
| Errors in Congruent trial | 0 (0) | 0 (0) | -- | -- |
| Errors in Incongruent trial | 0.21 (0.66) | 2.34 (4.29) | 3.86 | < 0.001 |
| Time difference in Congruent and Incongruent trials | 18.00 (7.56) | 31.37 (21.94) | 4.45 | < 0.001 |

*Note*. SST, Serial Seven Test; BBT, Box and Block Test; FD, frequency discrimination; MMSE, Mini-Mental Status Examination; MoCA, Montreal Cognitive Assessment

**Table S3**. Outcome measures at pre- and post-intervention in two groups.

|  | Cognitively Healthy (n=62) | | Cognitive Decline (n=44) | |
| --- | --- | --- | --- | --- |
|  | Pre-Intervention | Post-Intervention | Pre-Intervention | Post-Intervention |
| Measures | *M* (*SD*) | *M* (*SD*) | *M* (*SD*) | *M* (*SD*) |
| SST when tasked with BBT | 7.25 (4.73) | 8.37 (5.25) | 1.59 (2.49) | 1.63 (3.20) |
| BBT when tasked with SST | 55.51 (11.71) | 57.53 (11.49) | 45.14 (15.38) | 46.50 (14.32) |
| FD when tasked with BBT | 15.79 (2.41) | 16.29 (2.55) | 13.60 (4.36) | 15.46 (2.60) |
| BBT when tasked with FD | 68.34 (11.97) | 69.10 (10.88) | 58.81 (15.23) | 59.17 (13.15) |
| MMSE | 28.65 (1.46) | 28.60 (1.41) | 25.73 (2.78) | 25.30 (3.18) |
| MoCA | 28.15 (1.38) | 28.27 (1.42) | 21.48 (4.10) | 21.59 (4.67) |
| Digit-Symbol Coding | 12.98 (2.55) | 13.83 (2.71) | 10.37 (2.48) | 10.45 (2.39) |
| Word List |  |  |  |  |
| Immediate recall | 12.94 (2.64) | 13.90 (3.33) | 9.16 (2.48) | 9.31 (2.85) |
| Delayed recall | 12.53 (3.00) | 13.48 (3.10) | 9.48 (2.23) | 9.71 (2.32) |
| Stroop Color and Word Test |  |  |  |  |
| Errors in Congruent trial | 0 (0) | 0 (0) | 0 (0) | 0 (0) |
| Errors in Incongruent trial | 0.21 (0.66) | 0.16 (0.45) | 2.34 (4.29) | 2.49 (4.59) |
| Time difference in Congruent and Incongruent trials | 18.00 (7.56) | 14.18 (7.89) | 31.37 (21.94) | 28.76 (16.17) |

*Note*. SST, Serial Seven Test; BBT, Box and Block Test; FD, frequency discrimination; MMSE, Mini-Mental Status Examination; MoCA, Montreal Cognitive Assessment
